# Climbing favours the tripod gait over alternative faster insect gaits

**DOI:** 10.1038/ncomms14494

**Published:** 2017-02-17

**Authors:** Pavan Ramdya, Robin Thandiackal, Raphael Cherney, Thibault Asselborn, Richard Benton, Auke Jan Ijspeert, Dario Floreano

**Affiliations:** 1 Laboratory of Intelligent Systems, Institute of Microengineering, École Polytechnique Fédérale de Lausanne, Lausanne CH-1015, Switzerland; 2 Center for Integrative Genomics, Faculty of Biology and Medicine, University of Lausanne, Lausanne CH-1015, Switzerland; 3 Biorobotics Laboratory, Institute of Bioengineering, École Polytechnique Fédérale de Lausanne, Lausanne CH-1015, Switzerland

## Abstract

To escape danger or catch prey, running vertebrates rely on dynamic gaits with minimal ground contact. By contrast, most insects use a tripod gait that maintains at least three legs on the ground at any given time. One prevailing hypothesis for this difference in fast locomotor strategies is that tripod locomotion allows insects to rapidly navigate three-dimensional terrain. To test this, we computationally discovered fast locomotor gaits for a model based on *Drosophila melanogaster*. Indeed, the tripod gait emerges to the exclusion of many other possible gaits when optimizing fast upward climbing with leg adhesion. By contrast, novel two-legged bipod gaits are fastest on flat terrain without adhesion in the model and in a hexapod robot. Intriguingly, when adhesive leg structures in real *Drosophila* are covered, animals exhibit atypical bipod-like leg coordination. We propose that the requirement to climb vertical terrain may drive the prevalence of the tripod gait over faster alternative gaits with minimal ground contact.

## Abstract (web summary)

Numerous selective forces shape animal locomotion patterns and as a result, different animals evolved to use different gaits. Here, Ramdya *et al*. use live and *in silico* Drosophila, as well as an insect-model robot, to gain insights into the conditions that promote the ubiquitous tripod gait observed in most insects.

Since the pioneering photographic studies of Muybridge^1^, it has been widely appreciated that animals use distinct gaits at different locomotor speeds. These discontinuous shifts in leg coordination are hypothesized to minimize energy consumption by changing the number and relative timing of legs in motion^2^,^3^. For example, horses transition from a slow walk, lifting only one or two legs simultaneously, to a trot, canter and, finally, a fast gallop that further reduces the number of legs on the ground at any one time^4^. Above a certain threshold speed, most vertebrates use gaits that are characterized by having little to no ground contact during part of the stepping cycle and require dynamic stability to remain upright^5^.

Similar to vertebrates, insects also exhibit gait transitions as they increase locomotor speed, although it is less clear to what extent these transitions are true gaits^6^,^7^, or continuous changes over walking speed^8^,^9^,^10^. For example, *Drosophila melanogaster*, a popular model for studying insect locomotion^11^,^12^,^13^, transitions from a slow wave gait to a tetrapod gait and finally to a fast tripod gait^11^,^12^,^13^,^14^, always keeping at least five, four or three legs on the ground at a given time, respectively. During tripod ground locomotion, the front and rear legs on one side of the body move nearly synchronously with the middle leg on the other side. This tends to keep the animal's projected centre of mass (COM) within a three-point polygon of support formed by the legs: a defining feature of static stability^5^. Therefore, in sharp contrast to fast vertebrate running gaits that have at most one or two feet in contact with the ground, an overwhelming majority of running insects do not reduce the number of legs on the ground below three. Importantly, this is not an inherent difference between hexapods and quadrupeds: in rare cases, insects can have just two legs^15^, or no legs^16^ (that is, flight phases) on the ground during tripod running. More commonly, to further increase ground locomotor speed, insects increase stride length, increase stride frequency, invoke spring–mass dynamics^17^ and reduce duty factors^18^.

Fast gaits are critical for survival: they are used to hunt and to escape^19^. Therefore, despite the capacity for other gaits^7^,^12^, the ubiquity of the tripod gait across diverse insect species^14^ (for example, flies^12^, ants^20^, stick insects^8^, cockroaches^21^ and dung beetles^7^) suggests that it has been subject to selection as a means for achieving fast locomotion. However, the factors—ethological, biomechanical and/or developmental—causing the prevalence of this locomotor strategy over vertebrate-like gaits that minimize ground contact remain unknown. In nature, many small insects, including *Drosophila*, exhibit strong phototaxis and negative gravitaxis, compelling them to navigate and seek higher altitudes^22^ by climbing up obstacles in their surroundings. Therefore, one long-standing but untested hypothesis for why the tripod gait is so pervasive is that it allows insects to rapidly traverse challenging terrain, such as vertically oriented vegetation, without falling off^23^,^24^.

It is not yet possible to test this hypothesis by changing the gaits of real insects or by measuring the ancestral origins of extant locomotor behaviours. Therefore, computational approaches can be used to address experimentally intractable biological questions^3^,^25^,^26^,^27^,^28^,^29^. To investigate factors favouring the prevalence of the insect tripod gait, we discovered fast locomotor gaits for an *in silico* insect model using an optimization algorithm (Particle Swarm Optimization (PSO)^30^). Gaits can be characterized by their footfall patterns (for example, tripod or tetrapod), duty factors (above 0.5 for walking or below 0.5 for running^4^) and ground stability (static or dynamic). Here we focused on footfall patterns, as we were interested in understanding why insects rely on the tripod rather than alternative three-legged or even dynamically stable two-legged gaits during fast locomotion.

We find that the classic tripod gait is uniquely optimal for fast upward climbing using leg adhesion. It is also strongly favoured during downward and sideways climbing. However, this is not due to adhesion alone: a variety of other gaits are also optimal for fast ground walking with leg adhesion. By contrast, when optimizing for rapid ground locomotion in the absence of adhesion, novel dynamically stable two-legged gaits emerge. These bipod gaits are similar to the vertebrate running trot and are faster than the tripod gait in the insect model and in a hexapod robot. Intriguingly, when the structures subserving leg adhesion are blocked in real *D. melanogaster*, flies abandon the tripod gait and instead exhibit atypical bipod-like leg coordination. These data suggest that the prevalence of tripod locomotion in insects—over faster, vertebrate-like gaits with minimal leg–substrate contact—is related to the requirement to climb three-dimensional surfaces using leg adhesion.

## Results

### Gait optimization in an insect model

**Figure 1:**
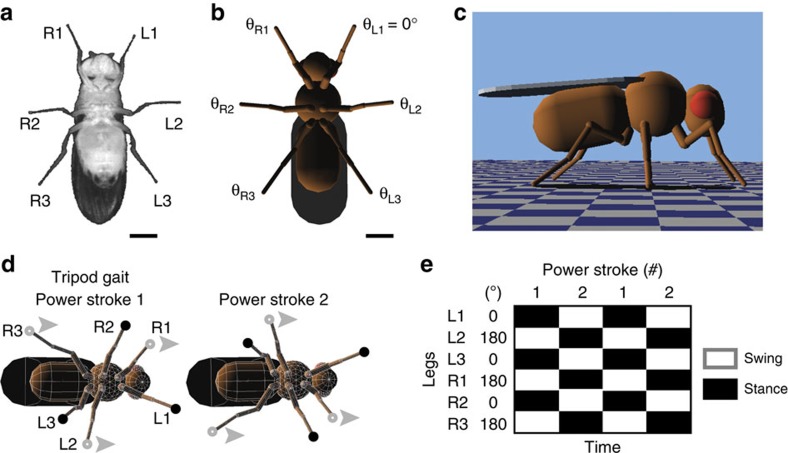
Gait optimization in an insect model. (**a**) A ventral view of a *D. melanogaster* female. Each leg is labelled as belonging to the right (R) or left (L) side and the prothoracic (1), mesothoracic (2) or metathoracic (3) leg pair. Scale bar, 0.4 mm. (**b**) A ventral view of the *in silico* insect model used in this study. A vector of five numbers encodes a single gait: each number represents a leg's phase of motion relative to the left front leg whose phase is fixed at 0°. Scale bar, 0.4 mm. (**c**) A side view of the insect model in its *in silico* environment. (**d**) The classic tripod gait has two power strokes per locomotor cycle. During each power stroke three legs are on the surface (stance phase, black circles), whereas the other three legs are off the surface (swing phase, grey circles). Grey arrowheads point in the direction of motion. (**e**) An idealized gait diagram of stance (black) and swing (white) phases for each leg during two cycles of tripod locomotion. The phase of motion for each leg is indicated. Each power stroke is numbered.

Our aim was to discover fast insect gaits for climbing or for ground locomotion. Therefore, we designed a physics-based insect model but minimized its complexity to reduce the computational cost of gait optimization. Specifically, we used the simulation engine, Webots^31^, to build a model based on the morphology and leg kinematics of *D. melanogaster* (Fig. 1a–c, Supplementary Fig. 1 and Supplementary Tables 1–5). To control each leg, instead of uisng complex neuromechanical methods^32^,^33^, we measured and reproduced periodic *D. melanogaster* leg motions during fast walking. In this way, we could isolate the contribution of gait on locomotor speed by varying the relative phases of motion of each leg, while keeping stride frequency and foot trajectories fixed.

In our model, a vector of five numbers encodes a single gait: each number represents a single leg's phase of motion relative to the left front leg, which is fixed at 0° phase (Fig. 1b). For example, the simplest way to generate a tripod gait in our model is to fix the front left (*θ*_L1_), middle right (*θ*_R2_), and rear left (*θ*_L3_) legs at a phase of 0°, while setting the remaining three legs to a phase of 180° (Supplementary Movie 1). The resulting gait has two power strokes per walking cycle (Fig. 1d) and can be characterized using a footfall or gait diagram that illustrates which legs are (stance) or are not (swing) in contact with the ground at each point in time (Fig. 1e). This gait produces ground reaction forces that rely on the front legs and, to some extent, the middle legs for propulsion (Supplementary Fig. 2a; it is noteworthy that diverse mechanisms for propulsion have been observed across insect species^34^,^35^,^36^).

Notably, these phase lags are used for open loop control of our model. By contrast, insects are thought to depend on a distributed control mechanism whereby the movements of each leg depend on their phases relative to those of other segmental legs (for example, hind leg movements take into account the current state of the middle legs)^8^,^9^,^13^,^14^,^23^,^37^,^38^,^39^. The advantage of our compressed method for encoding locomotor gaits with only five parameters is that it allows for a more rapid computational search for optimally fast gaits. Alternatively, if we had used existing neuromechanical insect models composed of many free parameters^32^,^33^,^40^,^41^, the time for gait optimization would be have been prohibitively long, it would have been more difficult to analyse the data and it would have been more challenging to extract general principles from the results.

During climbing, in addition to frictional forces, insects rely on adhesive forces^42^,^43^ generated by biomechanical specializations such as claws and pulvilli on their legs^21^,^44^,^45^. Frictional and adhesive forces differ in that they act in different directions—tangential and normal, respectively, to the contact surface—and therefore have different effects on the legs: friction reduces slipping, while adhesive forces act against lift-off of the legs. Therefore, in addition to frictional forces, for some experiments we added a contact-dependent adhesion force to the tips of the model's legs. The detailed physics of adhesion can vary depending on whether they originate from interlocking, capillary or dry mechanisms; however, at a higher level of abstraction these all generate normal forces that prevent the foot from lifting. As different adhesion mechanisms for vertical climbing can be modelled using a common template^21^, we did not incorporate fine-scale physical mechanisms for adhesion and substrate-release into our model.

We optimized our insect model's gait for forward velocity, resulting in gaits that generate straight locomotion. Optimization for energy efficiency (via measurements of cost of transport (COT)) or using a different optimization method (genetic algorithm) yielded similar results. We began each optimization experiment by generating a population of 50 insect models with random gaits (that is, random phases of motion for each leg). We then measured forward velocities for each model and used the fastest gaits—as well as the stochasticity inherent in PSO algorithms^30^—to define gaits to be tested in the next iteration of the algorithm. In this way, each model's forward velocity steadily improved, whereas the population's phase vectors converged over the course of 150 optimization iterations (Supplementary Fig. 3 and Supplementary Movie 2). After each optimization experiment, we identified and studied the single fastest gait found over all iterations.

### Tripod gaits are optimal for fast climbing with leg adhesion

Using this gait discovery approach, we first asked to what extent, if at all, the tripod gait would be discovered as fastest under different conditions. Specifically, we optimized gaits for (i) upward climbing, (ii) downward climbing (iii) or sideways climbing using leg adhesion, (iv) ground locomotion with leg adhesion or (v) ground locomotion in the absence of leg adhesion (*N*=15 each; Supplementary Data). These five conditions allowed us to measure the influence of travel orientation and/or leg adhesion on optimally fast gaits. For each experiment, gaits were classified based primarily on the model's footfall patterns, as even gaits that share similar leg motion phase vectors can behave differently depending on the model's orientation (vertical, or horizontal) and whether the model has leg adhesion. Across all five conditions we often discovered gaits with similar footfall patterns. Thus, we were able to classify most gaits as belonging to one of six categories (Supplementary Figs 4 and 5): the classic tripod gait (tripod-A), as well as alternative three-legged gaits (tripod-B and tripod-C) and two-legged gaits (bipod-A, bipod-B and bipod-C) that we later describe in more detail.

**Figure 2:**
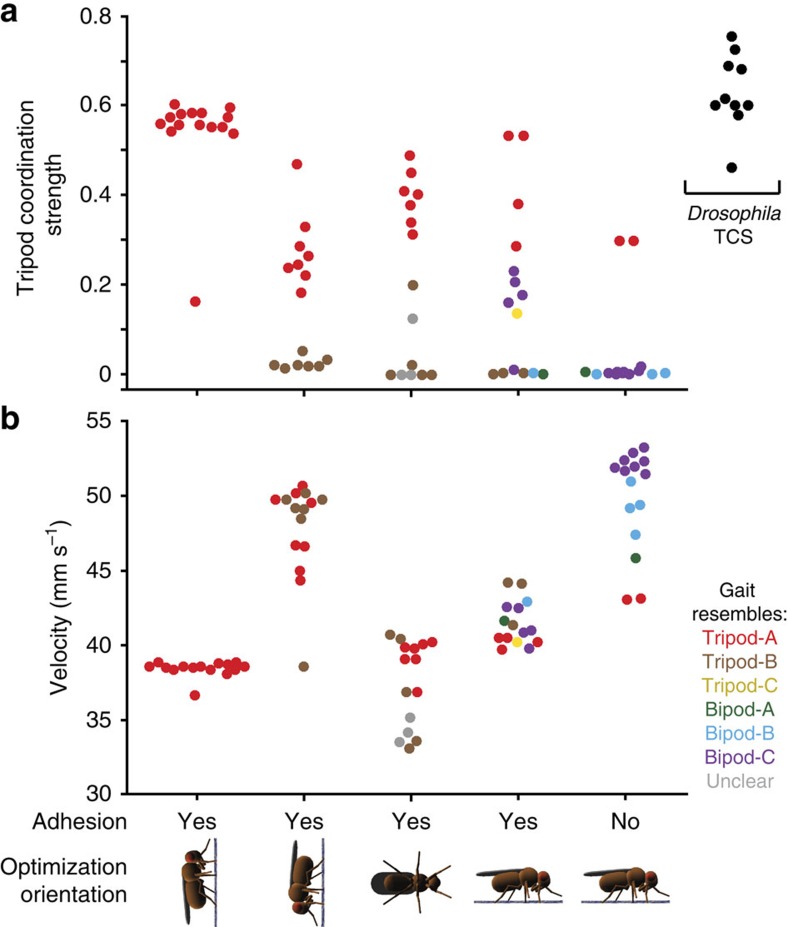
Tripod gaits are optimal for fast climbing using leg adhesion. Gaits were optimized for forward velocity, while climbing upward (left), downward (centre left) or sideways (centre) on a vertical surface using leg adhesion, walking on the ground with leg adhesion (centre right) or walking on the ground without leg adhesion (right). (**a**) TCS values indicating the degree of similarity to the classic tripod gait footfall diagram (tripod-A). *N*=15 for each condition. For comparison, TCS values for *D. melanogaster* during rapid, touch-evoked ground walking are shown on the far right (black, *N*=10). (**b**) The average velocity of each gait. Optimized gaits are color-coded by class. Data points are randomly scattered along the *x* axis for clarity. *N*=15 for each condition.

**Figure 3:**
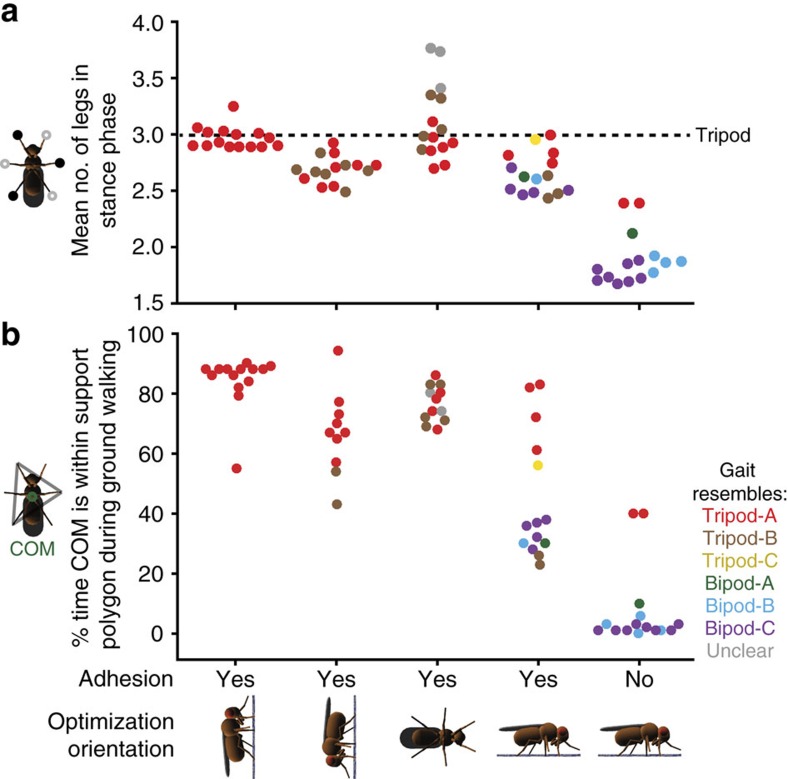
Dynamically stable bipod gaits are optimal for fast ground locomotion in the absence of leg adhesion. Gaits were optimized for forward velocity while climbing upward (left), downward (centre left) or sideways (centre) on a vertical surface using leg adhesion, walking on the ground with leg adhesion (centre right) or walking on the ground without leg adhesion (right). (**a**) The average number of legs in stance phase over five walking cycles. A dashed black line indicates three legs in stance phase as expected for the classic tripod gait. *N*=15 for each condition. (**b**) The percentage of time that the model's COM lies within a polygon of support delineated by each leg in stance phase when the gait is tested during ground walking. The level of adhesion is kept as during optimization. Optimized gaits are colour coded by class. Data points are randomly scattered along the x-axis for clarity. *N*=15 for each condition, except for vertical downward (*N*=11) and vertical sideways (*N*=12) locomotion. In these conditions several gaits were unable to support ground walking.

Gaits discovered as optimal for upward climbing using leg adhesion had high Tripod Coordination Strength (TCS, functionally similar to the quantification used in ref. 12) values (Fig. 2a, left), indicating that their footfall diagrams resemble that of the classic tripod gait (Fig. 1e). These values were only slightly lower than those measured for real *D. melanogaster* during touch-evoked fast locomotion^46^ (Fig. 2a, far right, ‘*Drosophila* TCS'; *P*=0.004, Wilcoxon's rank-sum test). Moreover, nearly all of the discovered gaits closely resembled one another (Supplementary Movie 3) and were classified as tripod-A, as their footfall patterns were quite similar to the classic tripod gait (Supplementary Fig. 4c). One gait had a low TCS (=0.16, experiment 5) and was also the slowest (Fig. 2b, left). Upward climbing gaits had on average three legs on the ground at any one time (Fig. 3a, left), forming a polygon of support within which the model's COM (projected normal to the surface) would rest when used for ground locomotion (Fig. 3b, left).

Interestingly, when optimizing for downward and sideways climbing using leg adhesion, in addition to the classic tripod gait, an alternative form of tripod coordination, the tripod-B gait also emerged (Supplementary Movie 3 and Fig. 2, centre left and centre). For the tripod-B gait, the front and middle legs on one side of the body move in near synchrony with the rear leg on the other side of the body (Supplementary Fig. 4a,b). This too yields an average of nearly three legs on the substrate at any given moment (Fig. 3a, centre left and centre) and the potential for static stability when used for ground locomotion (Fig. 3b, centre left and centre).

In contrast to climbing gaits, when optimizing for fast ground locomotion with leg adhesion, we discovered a variety of novel gaits (Supplementary Movie 3) that were as fast or even faster than the classic tripod gait (Fig. 2b, centre right) and could also have fewer legs in stance phase at any given moment (Fig. 3a, centre right). Taken together, these data demonstrate that a requirement to rapidly navigate vertically oriented terrain is sufficient to favour the classic tripod gait during optimization. Leg adhesion by itself has only a weak effect on the optimality of the classic tripod gait over alternative gaits.

### Bipod gaits for fast ground locomotion without adhesion

Large vertebrates typically do not depend on leg adhesion to locomote (but note the exceptional climbing abilities of smaller vertebrates such as geckos and squirrels). Instead, they rely on frictional forces to traverse the ground. By contrast, many insects use adhesive structures during ground locomotion. We hypothesized that this difference may have influenced the starkly different fast locomotor strategies used by vertebrates (dynamically stable running gaits) and insects (tripod gait). An interesting prediction of this hypothesis is that if insect gaits are optimized to locomote rapidly on the ground without leg adhesion, they might employ dynamically stable fast gaits instead of the tripod gait. To test this possibility, we optimized our insect model to generate gaits for rapid ground locomotion in the absence of leg adhesion. Indeed, we found that a large majority of optimized gaits bore little to no resemblance to the classic tripod gait (Fig. 2a, right, TCS ∼=0). Moreover, two gaits that could be classified as tripod-A (experiments 4 and 15) were also the slowest (Fig. 2b, right red circles and Supplementary Movie 3).

**Figure 4:**
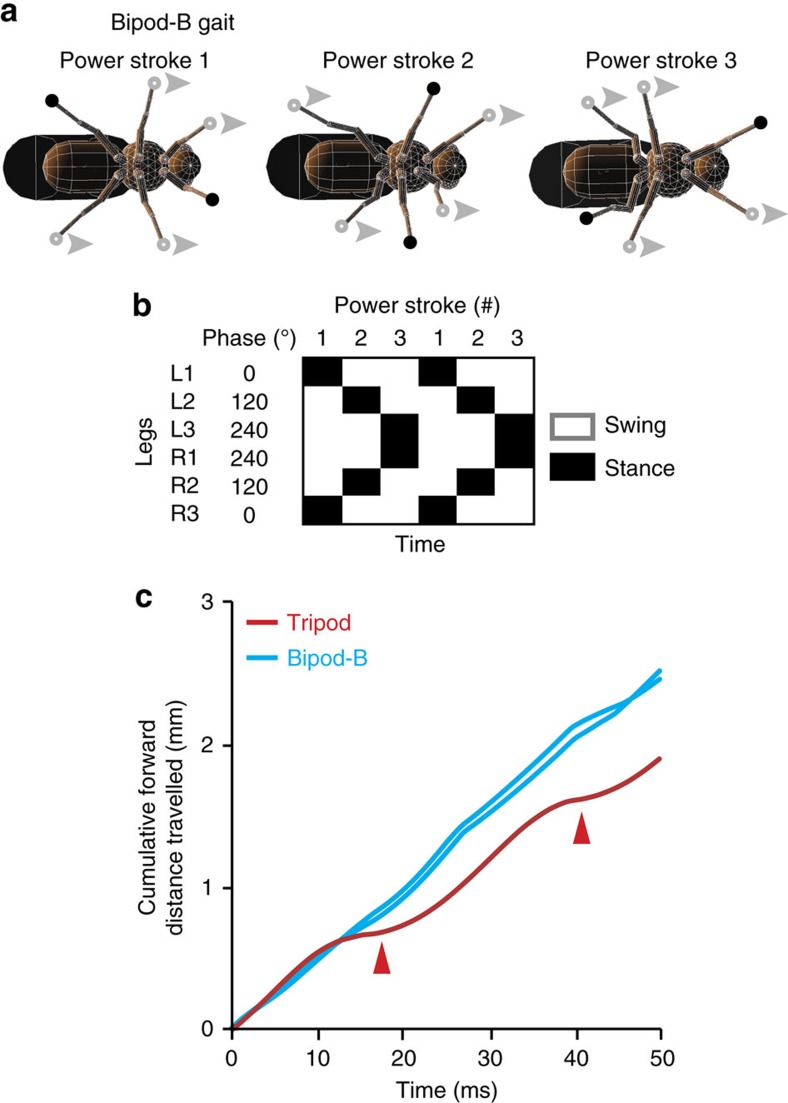
Bipod gaits generate three power strokes per walking cycle resulting in more continuous forward locomotion. (**a**) The bipod-B gait has three power strokes per walking cycle. For each power stroke two legs are on the surface in stance phase (black circles), while the other four legs are in swing phase (grey circles). Arrowheads show the direction of motion. (**b**) An idealized bipod-B footfall diagram showing stance (black) and swing (white) phases for each leg. The phase of motion for each leg is indicated. Each power stroke is numbered. (**c**) The cumulative forward distance travelled while upright without leg adhesion during a single walking cycle for the classic tripod (red) or for two optimized bipod-B (cyan) gaits. Red arrowheads indicate pauses between power strokes during tripod locomotion.

Instead, the fastest gaits had on average nearly two legs on the ground at any given moment (Fig. 3a, right) and low duty factors (<0.5, Supplementary Fig. 6e). Therefore, we named these bipod gaits. In many cases, during bipod locomotion a model's projected COM almost never lies within a polygon of support circumscribed by the legs (Fig. 3b, right) causing gaits to be statically unstable—like many fast vertebrate running gaits. In the American cockroach, during extremely fast tripod locomotion (>1 m s^−1^), aerodynamic forces lift the front and middle legs off the ground resulting, effectively, in two-legged running^15^. By contrast, in our insect model, two-legged bipod locomotion arises solely from leg coordination without a contribution from aerodynamics. During bipod-A and bipod-B locomotion, each front leg moves in near synchrony with the opposite rear leg and the middle legs move together (Fig. 4a). This generates three power strokes per locomotor cycle (Fig. 4a,b). Therefore, all else being equal (for example, same leg speeds), bipod gaits can generate more continuous and consequently faster forward locomotion than the tripod gait (Fig. 4c). Notably, although we did not optimize for it, bipod coordination is also energy efficient: It has a lower COT than the tripod gait during ground locomotion without adhesion (Supplementary Fig. 7, right).

### A bipod gait is faster than the tripod gait in a robot

**Figure 5:**
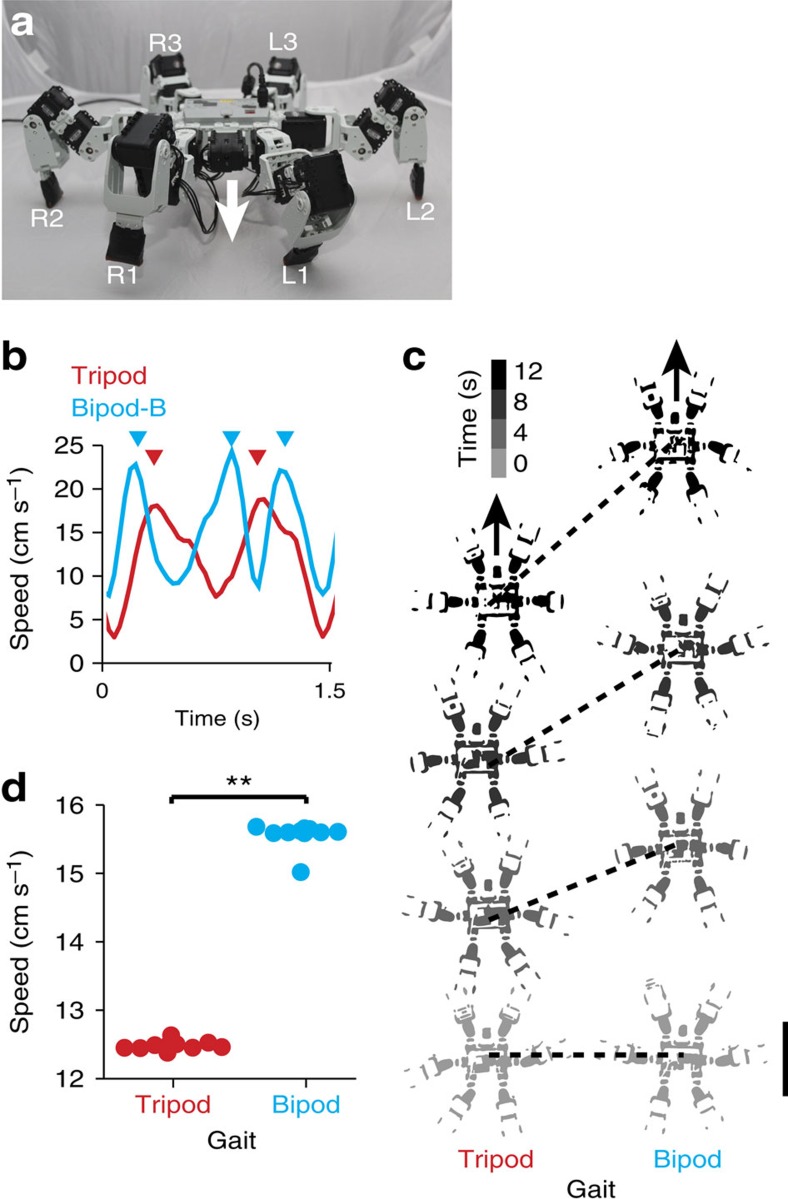
The bipod gait is faster than the tripod gait during ground locomotion in a hexapod robot. (**a**) A frontal view of the hexapod robot used. Each leg is labelled in white. A white arrowhead indicates the direction of heading. (**b**) The instantaneous speed of the robot during one walking cycle using the classic tripod (red) or bipod-B (cyan) gait. Red and cyan arrowheads mark peak speeds for the tripod and bipod-B gaits, respectively. (**c**) The robot's position at four 4 s intervals during tripod (left) or bipod-B (right) locomotion. Black dashed lines connect the robot's locations at corresponding time points. Black arrows indicate the direction of heading. Scale bar, 16 cm. (**d**) Average speed over 10 s for each gait (*N*=10 each). A double asterisk (**) indicates that *P*<0.001 for a two-sample *t*-test.

*In silico* findings can be sensitive to simulation conditions and may fail to capture the complexities of the physical world^47^. As it is not yet possible to genetically reprogramme insect leg coordination, we used a hexapod robot to validate our finding that bipod locomotion is faster than tripod locomotion. This also allowed us to explore whether our newly discovered bipod gaits could be used to effectively control hexapod ground robots. First, we transferred the classic tripod (tripod-A) gait and the bipod-B gait to a robot (Fig. 5a) using an inverse kinematic approach. In this way we could map the trajectories of the tips of the model's legs onto the tips of the robot's legs (Supplementary Fig. 8 and see Methods). We found that, as for the model, the robot produced two power strokes per walking cycle using the tripod gait (Fig. 5b, red) and three power strokes using a bipod gait (Fig. 5b, cyan). Remarkably, although it is profoundly morphologically different from the insect model (for example, size discrepancies and different degrees of freedom for each leg), the robot is also nearly 25% faster when using the bipod gait rather than the tripod gait (Fig. 5c,d, *P*<0.001, two-sample *t*-test and Supplementary Movie 4). These data confirm that a bipod gait is indeed faster than the classic tripod gait during ground walking in the absence of leg adhesion and that these gaits can be used to control fast locomotion in hexapod ground robots.

### Atypical bipod-like leg coordination in *D. melanogaster*

Despite being faster than the tripod gait, our dynamically stable bipod gaits are, to the best of our knowledge, not used by insects. This might be due to inherent neural or biomechanical constraints on limb control, that is, during fast locomotion, insects might be incapable of synchronizing their middle (mesothoracic) legs and synchronizing their contralateral front (prothoracic) and rear (metathoracic) legs. We define these kinds of novel leg synchronization as ‘atypical bipod-like leg coordination' to distinguish them from bipod gaits. Importantly, by this definition, atypical bipod-like leg coordination can occur even when more than two legs on the ground at one time. Because, in our model, removing leg adhesion led to a reduction in tripod gaits and enrichment in bipod gaits (Fig. 2, comparing ground locomotion with and without adhesion), we wondered how removing leg adhesion might influence fast locomotor gaits in real *D. melanogaster*.

**Figure 6:**
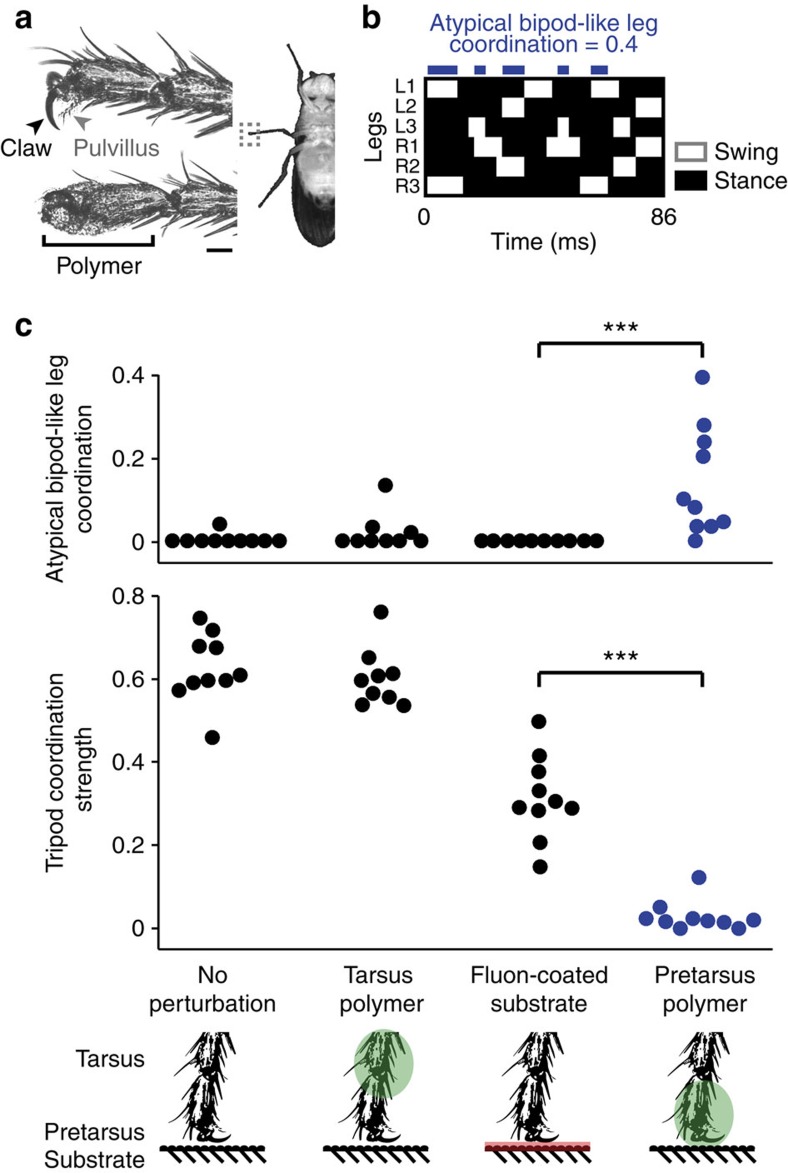
Blocking leg adhesion in *D. melanogaster* abolishes the tripod gait and uncovers the potential for atypical bipod-like leg coordination. (**a**) The pretarsus, the distal-most segment of the *D. melanogaster* leg (grey dashed box, right), houses a claw (black arrowhead) and pulvillus attachment pad (grey arrowhead), which are used to adhere to surfaces (left, top). We used a ultraviolet-curing polymer to cover pretarsal adhesive structures (left, bottom). Scale bar, 40 μm. (**b**) Footfall diagram for a fly walking with polymer coating on each pretarsus. Contact with the ground during stance phase (black) and no ground contact during swing phase (white) are indicated for each leg over time. Blue blocks indicate periods of atypical bipod-like leg coordination. This animal exhibits atypical bipod-like leg coordination 40% of the time. (**c**) Atypical bipod-like leg coordination (top) and TCS (bottom) for unperturbed flies (left), flies with polymer on each tarsus (green, middle-left), flies walking on a Fluon-coated substrate (pink, middle-right) or flies with polymer on each pretarsus (green, right). *N*=10, 9, 10 and 10 flies, respectively. A triple asterisk (***) indicates that *P*<0.001 for a Wilcoxon's rank-sum test.

To impair leg adhesion, we covered the claws and pulvilli of each leg with an ultraviolet curable, hard polymer (Fig. 6a). We then elicited a rapid walking response through gentle mechanical stimulation of the wings or abdomen^46^. Animals did not switch to dynamically stable bipod gaits with two legs on the ground in response to this perturbation. In fact, they often had four legs on the ground (Fig. 6b). However, these gaits did exhibit atypical bipod-like leg coordination: footfall patterns showed synchronized movement of the middle legs with one another and synchronized movement of contralateral front and hind legs with one another (Fig. 6b,c, top panel, right). Concomitantly, there was a nearly complete loss of TCS (Fig. 6c, bottom panel, right). By contrast, control animals without any perturbation or with a polymer coating on the more proximal tarsal segments (that is, leaving adhesion by the claws and pulvilli intact) did not exhibit atypical bipod-like leg coordination (Fig. 6c, top panel, left and centre left). Instead, they used gaits exhibiting normal, high TCS values (Fig. 6c, bottom panel, left and centre left).

One potentially trivial explanation for these results is that animals whose adhesive leg structures are covered simply slip and are unable to coordinate their limbs in any meaningful way. To examine this possibility and to more generally test the role of slipping on fast locomotor gaits, we studied flies walking rapidly on a surface coated with Fluon. Fluon coating lowers the coefficient of friction^48^ and also blocks claw and adhesive pad contact with the underlying substrate^49^. This causes slipping, making it very difficult for insects to adhere to surfaces, and preventing climbing^50^,^51^. We measured similar coefficients of static friction, *μ*_s_, for unperturbed animals on a Fluon-coated surface (*μ*_s_=0.84±0.13) as for animals with polymer coating on their distal tarsal segments on an uncoated surface (*μ*_s_=0.83±0.04).

Atypical bipod-like leg coordination was completely absent in animals walking rapidly on Fluon-coated surfaces (Fig. 6c, top panel, centre right, *P*<0.001 for a Wilcoxon's rank-sum test when compared with Pretarsus polymer experiments). Moreover, although reduced, the gaits of flies walking on Fluon could still have high TCS values (Fig. 6c, bottom panel, centre right, *P*<0.001 for a Wilcoxon's rank-sum test compared with Pretarsus polymer coating). These results reveal that atypical bipod-like leg coordination does not emerge simply because flies are slipping on the ground. Instead, leg adhesion structures and/or their associated sensory feedback are likely to play an important role in determining which gaits are used during fast ground locomotion. When leg adhesion structures are blocked, flies replace tripod gaits with alternative gaits including those with atypical bipod-like leg coordination.

## Discussion

In this study we asked which conditions might have led to the near universality of the tripod gait as a fast locomotor strategy among insects^7^,^8^,^12^,^14^,^20^,^21^. We used an optimization algorithm to discover fast locomotor gaits for a simulated insect model. Our modelling efforts were focused on optimizing the relative phases of motion for each leg: defining features of an animal's gait that are under direct control of the nervous system^52^,^53^. We did not model limb compliance and dynamical effects—such as spring-mass dynamics with in-phase transitions of energy between gravitational and kinetic energy—that are well established as important for fast insect gaits^15^,^18^ but independent of leg coordination.

We found that the tripod gait systematically emerges as optimally fast for climbing up vertical surfaces. Tripod locomotion was also well represented among gaits that are optimal for fast downward and sideways climbing. By contrast, diverse gaits were optimal for flat ground locomotion with leg adhesion. Among these, the tripod gait was not the fastest. These data support the possibility that the tripod gait may be favoured by insects because it permits rapid navigation of three-dimensional terrain. At first glance, this result seems rather intuitive: three-legged gaits form a closed polygon of attachment and allow an animal to pause in mid-stride without falling or swinging from vertically oriented surfaces. However, the tripod-B gait—an alternative three-legged gait that would not as strongly satisfy the requirements for static stability on the ground—also emerged as optimal for fast downward and sideways climbing. Therefore, upward climbing probably has more stringent requirements for achieving a good balance between speed and stability that may uniquely be satisfied by using the classic tripod gait.

Contrasting with optimal gaits for rapid climbing, we found that dynamically stable, two-legged gaits are optimal for fast ground locomotion in the absence of leg adhesion. These novel bipod insect gaits resemble the quadruped running trot^4^, a gait used by large vertebrates. Bipod ground locomotion is also faster than the tripod gait in a hexapod ground robot. Therefore, although the tripod gait may still be a favourable approach for controlling climbing robots^21^,^54^, bipod gaits confer significant speed advantages on the ground^55^,^56^. We hypothesize that bipod gaits are faster than tripod gaits because they generate one additional power stroke per leg motion cycle. However, this additional power stroke causes the model to use a statically unstable locomotor strategy. Although we emphasize the role of vertically oriented climbing in driving the optimality of tripod locomotion, adhesion alone might also serve to constrain available locomotor strategies: if adhesion is strong enough, a push-off force using three legs may be required to detach the other set of three legs from the substrate. In addition, isotropic push-off afforded by the tripod gait may be necessary to avoid toppling.

Computational models contribute to our understanding of biology by allowing us to test otherwise experimentally intractable questions. In this work, we aimed to disentangle the potential impacts of environmental (climbing) and biomechanical (leg adhesion) constraints on the optimality of extant insect locomotor strategies. Although simple models are powerful tools for testing mechanistic hypotheses in a systematic manner, their scope can be limited. For example, there are many ways to augment our insect model in future studies that would strengthen the model's ability to inform our understanding of insect locomotion. First, our model's position (P) controller currently has a high gain that generates only limited limb compliance. In future work, the model's limbs might be made more compliant by decreasing P gain and/or increasing joint elasticity^57^,^58^. Second, insects come in a variety of sizes and morphologies^59^. Although we obtained similar results using models that are several orders of magnitude larger than our original model (Supplementary Fig. 9 and Supplementary Movie 5), by testing models with a variety of body shapes we might also gain insight into the relationship between morphology and optimal locomotor strategies^20^. Finally, the details of leg adhesion can vary across species^44^,^60^, suggesting another important property of the model that may be modified to test its influence on gait optimality.

In line with a potentially critical role for adhesion, in our model, the absence of adhesion led to an enrichment of bipod gaits in place of tripod gaits. When we covered leg adhesion structures in real *D. melanogaster*, flies also abandoned the tripod gait in favour of gaits that exhibit synchronization of the middle legs and of the contralateral front and rear legs. This is notable, as middle leg synchronization is normally never observed in the fly. Although front and rear leg synchronization can be seen during slow *D. melanogaster* walking^11^,^12^, it is absent when animals generate rapid locomotion. These instances of what we refer to as ‘atypical bipod-like leg coordination' can have more than two legs on the ground and are therefore quite different from the dynamically stable bipod gaits discovered during insect model optimization. However, these kinds of changes in leg coordination might represent early adaptations to new environments that may ultimately become fixed. For example, dung beetles and water striders traverse sand and water surfaces, respectively. To do this, they use unique gaits for which the middle legs are synchronized^7^,^61^. This impressive capacity for flexible leg coordination suggests that neural and biomechanical constraints may not shape the locomotor strategies of insects as powerfully as the need to solve specific challenges posed by the environment.

## Methods

### Insect model morphology

We designed an insect model using Webots 6.4.4 (ref. 31) (Cyberbotics Ltd, Lausanne Switzerland), a three-dimensional, physics simulation environment built on top of the Open Dynamics Engine (ODE). Using this software, solid geometric objects can be combined to build structures of arbitrary shape and actuated by simulated motors (see description below). We used Webots rather than a custom-designed physics engine and simulation environment to facilitate the reproduction and extension of our results by other researchers.

To develop our model we combined published anatomical information^62^,^63^ with microscope (Leica Microsystems, Wetzlar, Germany) images of 2 days post-eclosion (dpe) awake and anaesthetized female *D. melanogaster* of the *Canton S* background raised at 25 °C. As specimens were of variable size, we normalized measurements of each body and leg segment using the length of the thoracic segment as a reference^64^. These values were then used to determine the size of our insect model. The mass of the model was also based on the average weight of 2 dpe *D. melanogaster* females (0.85 mg).

The head, thorax and abdomen of the model together comprise one rigid body. However, each component has its own homogeneous mass. This determines the mass and inertia of the rigid body as a whole. The head and abdomen are modelled as rigid capsules, while the thorax is modelled as a rigid sphere. Each of the legs has six degrees of freedom. Each degree of freedom is implemented as a hinge joint. There are three hinge joints in series (that is, overlaid joint axes) at the body–coxa junction, one hinge joint at the coxa–femur junction, one hinge joint at femur–tibia junction and one hinge joint at tibia–tarsus junction. The segments of each leg are modelled as rigid capsules. The pretarsus, which is connected to the tarsus, is modelled as a rigid sphere.

### Leg motion kinematics

We use Webots position-controlled (internal P-controller) motors at each of the leg joints. These use angular position as a reference and determine the motor torque based on the P-controller. We used position control with strictly imposed leg movements since it simplifies the optimization landscape compared with more complex simulations that include muscle dynamics. Each of the leg joints is implemented as a servo node (that is, a hinge joint with a rotational motor). The motor is operated in position control with a P-controller of constant gain. Based on the target reference position, the P-controller computes the current velocity and the necessary torque, which is then applied directly at the joint by the physics simulator. At each simulation step, the P-controller computes the current velocity *ν*_*c*_ as in equation (1):


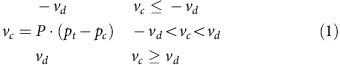


where *ν*_*c*_ is the current servo velocity, *P* is the P-control parameter specified in the control P-field, *p*_*t*_ is the target position of the servo (predefined), *p*_*c*_ is the current servo position, *ν*_*d*_ is the desired velocity as specified by the maxVelocity field. This is a standard implementation of the ODE/Webots P-controller. We did not limit acceleration.

ODE joint motors have two essential parameters: velocity and maximum torque. The maximum torque is predefined for each joint, whereas the velocity is computed by the P-controller. The effective torque that has to be applied is then computed such that the desired velocity is reached within one time step.

We defined the range of motion for each leg joint based on observations of freely walking *D. melanogaster* using high-speed videography (Gloor Instruments, Uster, Switzerland) and by referring to previous studies on insect locomotion and leg organization^38^,^62^,^63^. During ground locomotion, gravitational surface friction is the main interaction force. The movements of the legs relative to the head–thorax–abdomen rigid body are the same for all gaits. Based on observations of *D. melanogaster* walking in high-speed videos, the movements of each leg were preprogrammed as sinusoidal joint angle movements within an observed range of motion and fixed phase lags between the motions for each joint (Supplementary Fig. 1 and Supplementary Movie 1). Although the movements of the legs are fixed, their duty factor—how long a foot is in contact with the ground—varies and is free to emerge during our gait optimization. This is because foot contacts depend on the roll, pitch and elevation movements of the whole body, which vary over time and depend on the gait. Similarly, the stride length (progress per cycle) emerges during optimization. However, the stride frequency is kept constant at 20 Hz, a stride frequency that we measured from rapidly walking flies. For larger models (25 and 250 mm), stride frequencies were scaled based on the Froude number. Specifically, 20 Hz in the 2.5 mm model corresponds to 6.32 Hz for the 25 mm model and 2 Hz for the 250 mm model. However, to have an integer number of simulation steps, a frequency of 5 Hz was used for the 25 mm model.

We computed the frictional forces with the surface according to equation (2):


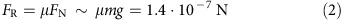


where the friction coefficient *μ*=0.1, leg mass *m*=1.42.10^−7^ kg and gravitational acceleration *g*=9.81 m s^−2^. The ODE uses a simple Coulomb friction model. Specifically, we use symmetric coulomb friction in Webots and default values for bounce and bounceVelocity. The bounce parameter defines the type of collision (1: elastic collision, <1: inelastic collision). Our contactProperties node was set to the following—[coulombFriction=4; bounce=0.5; bounceVelocity=0.01; forceDependentSlip=0]. A friction pyramid (approximation of a friction cone) is used to determine when slipping begins. We use a friction coefficient of *μ*=4. This represents an upper bound rather than the classical coulomb friction coefficient. A contact point with a ground reaction force that lies within the friction cone leads to a non-slipping contact. When the ground reaction force moves out of the friction cone, a slipping contact is established. In Webots/ODE the friction cone is approximated by a friction pyramid (that is, apex at the contact point, axis aligned with the normal force direction, base defined by two orthogonal tangential directions of ground plane). To compute the effective friction force, the ODE first assumes that the contact is frictionless and computes the resulting normal force *F*_*n*_. Based on this, the maximum frictional force in either of the two tangential directions is computed as 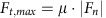. The ODE then continues to solve the system based on these limits for two cases:

1. Static friction: the ground reaction force lies inside the friction pyramid. Therefore, the frictional force will be computed to compensate for tangential forces.
2. Dynamic friction: the ground reaction force lies outside the friction pyramid. Therefore, the frictional force is computed as *F*_*t,max*_.

In addition to a universal frictional force, a leg-adhesion force is present on the pretarsus of each of the model's legs to mimic adhesive structures (claws and pulvilli). The adhesion force is applied during sticking and sliding conditions as soon as there is contact (collision) between the foot and the substrate plane. The strength of this force was determined by measuring how many pretarsi are required for *D. melanogaster* to suspend inverted from a cotton substrate for more than 1 s. In our experiments, one pretarsus was sufficient for substrate adhesion (two pretarsi: 10/10 flies hanging >1 s; one pretarsus: 11/11 flies hanging for >1 s; no pretarsi: 0/10 flies hanging for >1 s). Therefore, in our insect model, a minimal adhesive force, *F*_ad_, equivalent to that required for a single contact point/pretarsus to suspend the model in an inverted orientation is considered a 100% adhesion level. For our experiments we used 200% adhesion, as this was the minimal amount required for gait optimization to be successful in all possible travel orientations (for example, vertical). Although leg adhesion forces have not been formally measured for *D. melanogaster*, this is likely to be a lower bound based on studies of other species^65^,^66^. Adhesive forces are implemented as constant normal forces acting on the pretarsus when it is in contact with the substrate. As the normal force plays a role in the friction model, the adhesion force also has an impact on friction. For example, with 200% adhesion, each leg that is in contact with the ground experiences an additional normal force due to adhesion that corresponds to twice the weight of the insect model. As explained above, this normal force, *F*_*n*_, represents an upper bound inside the friction pyramid. The effective tangential friction force will be much smaller and just enough to ensure static contact. In other words, the adhesive force modifies the friction pyramid criterion to make tangential slipping much harder.

It is noteworthy that the issues concerning a closed kinematic chain are resolved for our model. A kinematic chain is a series of rigid bodies connected via joints whose movements are therefore coupled. A closed kinematic chain implies a series that contains at least two fixed joints, thereby creating a loop. When the model is using a tripod gait and three legs are in sticking contact with the ground, a closed kinematic chain is present with three fixed points/joints on the ground. Each leg has a predefined movement based on the model's kinematics, leaving us with three degrees of freedom for each leg's phase of motion. One might therefore expect that there are not enough degrees of freedom to achieve forward movement while using the tripod gait. However, the tripod gait can still be exploited in two ways. First, in the absence of adhesion, slipping (reduced normal force) is present. Therefore, legs in contact with the substrate may be not be in sticking contact, relaxing the constraints imposed by the closed kinematic chain. Second, in the presence of leg adhesion, the P controlled joint trajectories exhibit a minimal compliant behaviour that is sufficient to make the tripod gait possible. Evidence of our model's ability to overcome restrictions imposed by the closed kinematic chain can be seen in the results of our optimization experiments: the tripod gait emerges in each of the five conditions, both with and without adhesion.

We did not model air drag in our simulations. Although drag is an important factor for insect flight, we found that it is not nearly as relevant as frictional forces for walking. We computed drag forces for a single leg according to equation (3):


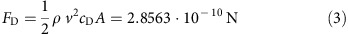


where *ρ*=1.225 kg m^−3^ (density of air), *c*_D_=1.05 (drag coefficient for a cube). The velocity *ν* was computed based on the frequency of leg motion and length of each leg: *ν*=2*πfL*=0.0628, m s^−1^, where *f*=20 Hz and *L*=0.0005, m. We considered the area of a leg as the drag area *A* where *A*=*d*.*L*=5.10^−8^ m^2^, where *d*=100 μm. Frictional forces are therefore three orders of magnitude larger and dominate drag forces. Similarly, if drag forces are calculated for the whole body, the velocity *ν*_c_=0.02 m s^−1^ (the approximate maximum walking speed of a fly) and the drag area *A*=0.0025^2^ m^2^ (the cross-sectional area approximated by the square of a fly's length). In this case *F*_D_=1.6·10^−9^ N and is still several orders of magnitude smaller than frictional forces.

### Gait optimization

We used PSO^30^, a stochastic optimization algorithm^47^, to discover gaits that optimize forward velocity under different adhesion and travel orientation conditions. We implemented PSO using the inspyred (inspyred.github.com) Python ( python.org) framework. Briefly, 50 candidate gaits (particles) were randomly initialized within a 5-dimensional search space of possible solutions. Each dimension represents one leg's phase of motion relative to the front left leg, which was fixed at 0° phase. During PSO, the phases of the five remaining legs could vary between 0° and 360°. For example, in this formulation a phase vector [L1=0°; R1=180°; L2=180°; R2=0°; L3=0°; R3=180°] defines the classic tripod gait, which we call tripod-A to distinguish it from alternative three-legged gaits.

Each particle was initialized with a random velocity that defined its movement within this search space during an iteration of the algorithm. Then, each particle's gait was simulated in the model. We measured its forward velocity over 0.5 s of simulated time (fitness). This allowed us to bias optimization for straight locomotion. Notably, we did not explicitly optimize for energy efficiency but nevertheless obtained more energy efficient gaits as a byproduct of speed optimization. For each iteration, particle positions were adjusted according to equations (4) and (5):


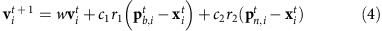



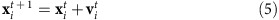


where 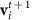 is the velocity at *t*+1 of particle *i*, 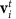
is the current velocity of the particle, 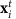
is the current position of the particle, 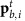 is the position of the personal best solution of particle *i*, 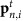
is the position of the neighborhood best solution, *r*_1_ and *r*_2_ are random numbers in the range [0,1], and the coefficients *w* (inertia weight), *c*_1_ (cognitive rate), and *c*_2_ (social rate) are fixed. For our simulation, we use the suggested^30^,^67^ values *w*=0.729, *c*_1_=1.49 and *c*_2_=1.49. In addition, we limited the maximum particle velocity to 0.4 (a fraction of the parameter space). The approach described here and implemented in inspyred is outlined in^67^. As our search space is periodic/circular (0=2*π*), we implemented a specialized bounding function using a custom PSO class that inherits the inspyred class and replaces the vector determination function. For each experimental condition, we ran 15 experiments with 50 particles each over 150 iterations. This number of iterations was chosen after assessing the convergence time of the optimization process. This number of experiments was chosen to best explore the search space of possible gaits while also limiting computing time.

We optimized for different geometric conditions (for example, ground and vertical locomotion) by changing the global direction of gravitational acceleration. If a model fell off of the substrate during vertical locomotion, the forward distance travelled before falling was taken into account when calculating speed rather than setting the fitness to zero. This procedure helped smooth the fitness landscape and assisted optimization.

### Gait classification

We classified optimized gaits based primarily on footfall patterns (as assessed using a gait diagram) and, to a lesser extent, phase vectors. Footfall patterns were emphasized over phase vectors, as gaits are highly dependent on leg adhesion conditions and locomotor orientation even for those with similar phase vectors (for example, tripod-A and bipod-C). Therefore, footfall patterns are more closely linked to the success or failure of a given gait than the underlying phases of motion for each leg. For the sake of completeness, we also present a quantification of the degree to which a gait's phase vector approximates the ideal phase vector for each class (tripod-A, tripod-B, tripod-C, bipod-A, bipod-B and bipod-C). An ideal phase vector for each gait class was determined by considering the average phases across all gaits jointly comprising a class and, whenever possible, by biasing these phases to be left/right leg symmetric. Then, the phase vector for each optimized gait was compared with this ideal phase vector of a given class to generate an error metric (*m*) according to equation (6):


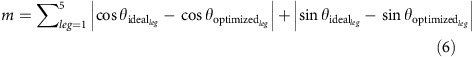


where *leg* is the leg being examined, *θ*_ideal_ is the phase vector for the ideal version of a given gait class and *θ*_optimized_ is the phase vector for the optimized gait being studied. After studying each optimized gait in this way, cases with high error values were re-examined to identify potentially incorrect classifications. Ultimately, however, ambiguities in classification between two potential gait classes were resolved by examining how well footfall diagrams for each gait resembled those of each gait class. Unique, unstructured (that is, asymmetric) gaits were classified as ‘unclear'.

### Gait analyses

We measured actual leg contacts with the surface (stance periods) to generate footfall/gait diagrams. To calculate the mean number of legs in stance phase, we averaged the number of legs in stance phase over multiple walking cycles. To measure duty factors, we averaged the relative amount of time that a given leg was in stance phase over five locomotor cycles. We measured ground reaction forces using touch sensors on each pretarsus. These sensors measure the three-dimensional contact force of the ball foot with the environment. The three-dimensional forces are first measured in the local coordinate frame of the pretarsus rigid body and then transformed to global coordinates by measuring the orientation of the pretarsus with respect to the global frame. We consider straight gaits, and therefore we can relate anterioposterior and mediolateral forces to x and y coordinates of the global frame. We smoothed noisy sensor measurements in post-processing.

As a measure of the potential for static stability, we asked whether the projection of the COM to the surface plane fell inside a convex support polygon formed by the foot endpoints in contact with the substrate^5^. This COM criterion can only relate to static stability for the ground case and not for vertical locomotion. Therefore, to measure the stability of gaits optimized during vertical climbing, we retested these gaits during ground locomotion with adhesion. In several instances we found that gaits optimized for vertical sideways and vertical downward locomotion were unable to support ground locomotion. These gaits failed in the first walking cycle and were excluded from subsequent analysis. We measured the percentage of time that body postures fulfilled this criterion to determine the overall stability characteristics of a particular gait. For each analysed gait the first locomotor cycle was omitted (to achieve steady state) and the remaining nine cycles were evaluated.

Calculating metabolic cost in biological systems is complicated for a number of reasons^68^. However, as a first approximation we equated mechanical energy as metabolic cost in our insect model. Specifically, as an estimate of energy consumption, we measured the COT, a dimensionless value, according to equation (7):


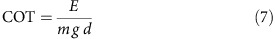


where *E* represents the energy needed to move the system along a distance *d*. *m* denotes the mass and *g* the gravitational acceleration. *E* is defined as the integral of power according to equation (9):


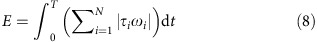


where *τ*_*i*_ and *ω*_*i*_ denote the applied torque and angular velocity at the *i*-th joint, respectively. *T* corresponds to the simulation time (0.5 s) and *N* is the number of motors across all the legs.

### Hexapod robot experiments

To test our *in silico* results in a physical system, we built a *Bioloid* hexapod robot (Robotis Inc., Seoul, Korea, http://en.robotis.com/index/product.php?cate_code=121010). This robot is 57 cm long from front leg tip to rear leg tip at full leg extension and weighs 1.9 kg. The morphology of the robot is quite different from the insect model since it is much larger and lacks a head, abdomen, and several leg segments. However, as morphologically diverse insects have similar footfall patterns we reasoned that these characteristics of locomotor gaits might be robust to morphological differences in our experiments as well.

To ensure adequate friction between the robot and the ground, a piece of latex with a static friction coefficient, *μ*, of 0.71 was bound to the tip of each leg. The robot has three leg degrees of freedom (one promotion/remotion and two flexion/extension) compared with the six degrees of freedom in the insect model (one rotation, one promotion/remotion and four flexion/extension). Therefore, to implement the model's cyclical motions for each leg, we discarded the rotation joint from the model, linked the promotion/remotion joints of the model directly to the robot and used an inverse kinematics approach to map four flexion/extension joints of the model to the robot's two flexion/extension joints.

To compute this mapping between the robot and insect model we first wrote a custom Python script to measure the joint angles of the model through time. With the known angles and segment lengths of our model (Supplementary Fig. 8b), we could identify the leg tip positions in their plane of motion by solving equations (9) and (10):


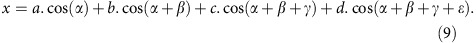



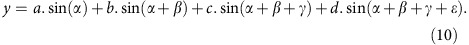


where *a*, *b*, *c* and *d* are the lengths of the model's leg segments (proximal to distal) and *α*, *β*, *γ* and *ɛ* are their respective joint angles. Using these data, our next goal was to find the angles, *λ* and *σ* that will place the tips of the robot's legs in the same position (*x*_1_, *y*_1_) as the model's legs. To do this we solved equations (11) and (12):


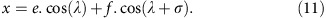



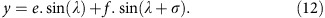


where *e* and *f* are the lengths of the robot's leg segments (proximal to distal), and *λ* and *σ* are their respective joint angles. Of the two solutions, we identified the one that was physically feasible in the robot. In addition, a bias was added to ensure that the model's range of promotion/remotion angles could be matched in the robot. To produce different gaits, as for the insect model, we shifted the relative phase of each leg's motion cycle. The resulting trajectories of the robot's foot tips are shown in Supplementary Fig. S6e.

We video recorded (Canon, Melville, NY, USA) the robot at 25 fps to quantify leg kinematics and speed. We then used custom Matlab scripts (The Mathworks, Natick, MA, USA) to track the motion of red markers on the leg tips, for leg kinematic measurements, or on the dorsal surface of the robot, for speed measurements. We performed ten experiments for each condition but found very few differences between experimental replicates. Data were analysed using the two-sample *t*-test, as they were normally distributed. We show all the raw data points for each experiment to illustrate the variance for each condition and that this variance is the same between compared groups.

### *Drosophila* experiments

Experiments were performed at 22 °C in the late afternoon Zeitgeber Time on 2–4 dpe female *D. melanogaster* (∼2.5 mm long and ∼0.85 mg) of the *Canton S* background raised at 25 °C on a 12 h light:12 h dark cycle. We filmed individual flies in a small Poly(methyl methacrylate) arena (3 cm × 3 cm) illuminated by a dim red ring light (FALCON Illumination MV, Offenau, Germany). We continuously acquired images at 500 fps using a high-speed video camera (Gloor Instruments). To motivate fast forward locomotion, we grazed the wings with a small metallic disc (1 mm diameter) to elicit an escape response. If a fly exhibited a long bout of straight locomotion (that is, without premature voluntarily stopping and without encountering the arena wall), a video was captured and manually analysed to measure stance and swing phases for each leg. This criterion for data inclusion was pre-established. No randomization or blinding was performed. Fast gaits are typically very consistent across animals. Nevertheless, we performed multiple replicates (*N*=9–10) for each condition to account for trial-to-trial differences in ultraviolet polymer coating and inter-animal variability. Data were typically not normally distributed. Therefore, we used a Wilcoxon's rank-sum test for statistical comparisons. We show all raw TCS and atypical bipod-like leg coordination strength data points for each experiment to illustrate the variance for each condition and that this variance is not the same between compared groups. This difference is due to the floor effect on TCS and atypical bipod-like leg coordination metrics.

For polymer coating, flies were first briefly anaesthetized with CO_2_. We then placed a small drop of ultraviolet-curing glue (Ivoclar Vivadent AG, Schaan, Principality of Liechtenstein) on the pretarsus or on the tarsus of each leg using a fine hair or tungsten wire. The polymer was then hardened by 20 s exposure to ultraviolet light. Flies were allowed to recover for 1–2 h in humidified 25 °C incubators. Before behaviour experiments, we confirmed the absence of adhesion by testing if flies could hang vertically on the smooth walls of a plastic vial. For substrate coating experiments, we dried a layer of Fluon (Whitford GmbH, Diez, Germany) on the walking surface. We also clipped each fly's wings to permit visualization of every leg from a dorsal perspective.

To measure the static coefficient of friction of animals, we placed flies (with or without polymer coating) on a horizontal surface (with or without Fluon coating) and measured the tilt angle, *θ*, at which animals began to slide. The static coefficient of friction, *μ*_s_, was then calculated according to equation (13):


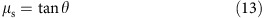


We analysed locomotor gaits using several metrics of leg coordination. First, we used a TCS metric functionally similar to one used previously^12^ to compare measured gaits to the classic insect tripod gait. Specifically, after initiating fast touch-evoked escape walking, we measured the first frame during which the right front leg was in stance phase and, following three walking cycles, the final frame during which the front right leg was in swing phase. This period of three walking cycles was deemed *t*_1_. Then, we measure the proportion of time, *t*_2_, during this period during which an animal is in a tripod stance (only R1, L2, R3 are in stance or only L1, R2, L3 are in stance). TCS values are the ratio *t*_2_*/t*_1_. Similarly, we quantified atypical bipod-like leg coordination by measuring the proportion of time that the contralateral front and rear legs (L1 and R3, or L3 and R1) or middle legs (L2 and R2) moved synchronously in swing phase. These kinds of leg synchronization are not normally observed during fast *D. melanogaster* locomotion^11^,^12^.

### Data availability

Gait data are provided in Supplementary Data. The remaining Webots, robotics and *D. melanogaster* data sets and code are available from the corresponding author on reasonable request.

## Additional information

**How to cite this article:** Ramdya, P. *et al*. Climbing favours the tripod gait over alternative faster insect gaits. *Nat. Commun.* **8,** 14494 doi: 10.1038/ncomms14494 (2017).

**Publisher's note:** Springer Nature remains neutral with regard to jurisdictional claims in published maps and institutional affiliations.

## Supplementary Material

Supplementary Information Supplementary Figures and Tables

Supplementary Movie 1 Comparison of *Drosophila melanogaster* and the insect model. (top) Side view of a female *D. melanogaster* walking using a tripod gait.(bottom) Side view of the insect model walking using a tripod gait. Movie is slowed down to 0.05x real-time.

Supplementary Movie 2 Gaits improve over the course of optimization. A ventral view of the insect model walking using the best gait identified for iterations 1, 2, 5, 9, 13, 19, 58, and 144 of a single optimization experiment. Optimization was performed for upward climbing using leg adhesion (experiment #10). Red circles indicate that a leg is in contact with the substrate. Movie is slowed down to 0.05x real-time.

Supplementary Movie 3 Gaits optimized for upward, downward, and sideways climbing using leg adhesion as well as ground walking with and without adhesion. A ventral view of the insect model walking with each of 15 optimized gaits for each condition. Gravity exerts a force on the model, pulling it backward, forward, rightward, and toward the substrate, respectively. Red circles indicate that a leg is in contact with the substrate. Movie is slowed down to 0. 05x real-time. The insect model is 2.5 mm long and is walking at 20Hz.

Supplementary Movie 4 Comparison of a hexapod robot walking on the ground without adhesion using either a tripod or a bipod gait.

Supplementary Movie 5 Gaits optimized for upward 132 climbing using leg adhesionand ground walking without adhesion in a 25 mm, or 250 mm long insect model. Aventral view of the insect model walking at 5Hz (25 mm model), or 2Hz (250 mm model)with each of 15 optimized gaits. Gravity exerts a force on the model, pulling it backward (upward climbing), or toward the substrate (ground walking). Red circles indicate that a leg is in contact with the substrate. Movie is slowed down to 0.1x (25 mm model), or 138 0.5x (250 mm model) real-time.

Supplementary Data 1 Raw data for insect model gait optimization and hexapod robot experiments.
