# Supplementary material for: Climbing favours the tripod gait over alternative faster insect gaits
Source: Nat Commun. 2017 Feb 17;8:14494. doi: 10.1038/ncomms14494 (PMC5321742; doi:10.1038/ncomms14494)
Supplement: Supplementary Information — Supplementary Figures and Tables [file ncomms14494-s1.pdf]

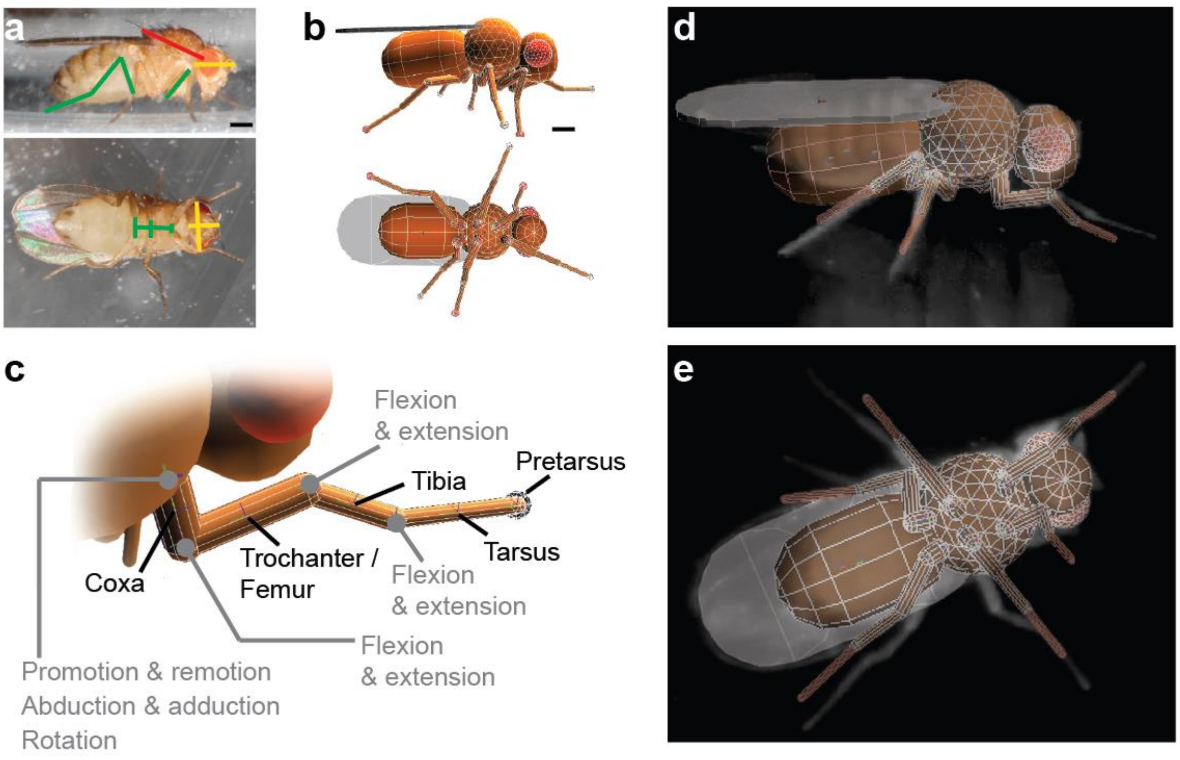

Supplementary Figure 1 | An insect model based on *Drosophila melanogaster*. (a) Side and ventral images of adult female flies used to calculate the sizes of body and leg segments. Scale bar is 0.3 mm. Green, yellow, and red lines illustrate examples of leg, head, and thoracic measurements, respectively. **(b)** Corresponding side and ventral views of the insect model. Scale bar is 0.3 mm. **(c)** Image of the model's front right leg. Leg segments and the degrees of freedom for each joint are labeled in black and grey, respectively. **(d, e)** Sample high-speed video images of *D. melanogaster* walking (grey) are overlaid by semi-transparent images of the insect model as seen from the side **(d)** or from below **(e)**.

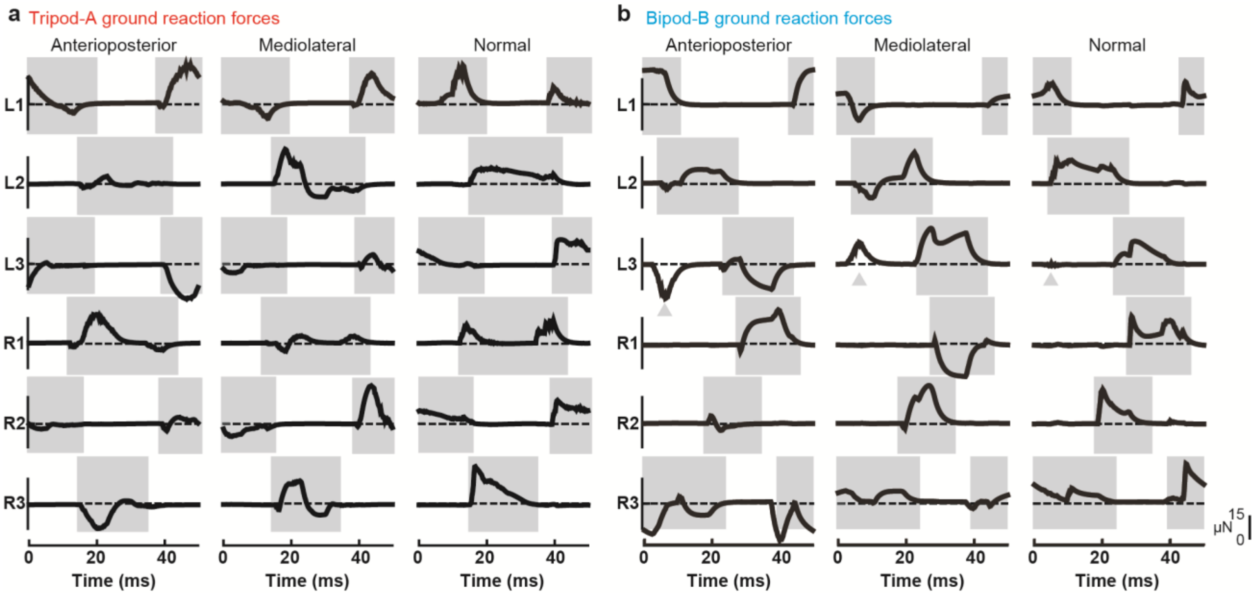

Supplementary Figure 2 | Ground reaction forces for the insect model. Ground reaction forces (GRF) for ideal **(a)** tripod-A and **(b)** bipod-B gaits. Shown are GRFs for each leg along the anterioposterior axis (left; positive values indicate GRFs pointing in the forward direction – propulsive forces), mediolateral axis (middle; positive values indicate GRFs pointing medially), and normal axis (right; positive values indicate GRFs pointing away from the surface). Gray boxes highlight stance epochs for each leg during tripod-A and bipod-B locomotion. Gray arrowheads indicate an instance of ground contact with minimal normal force.

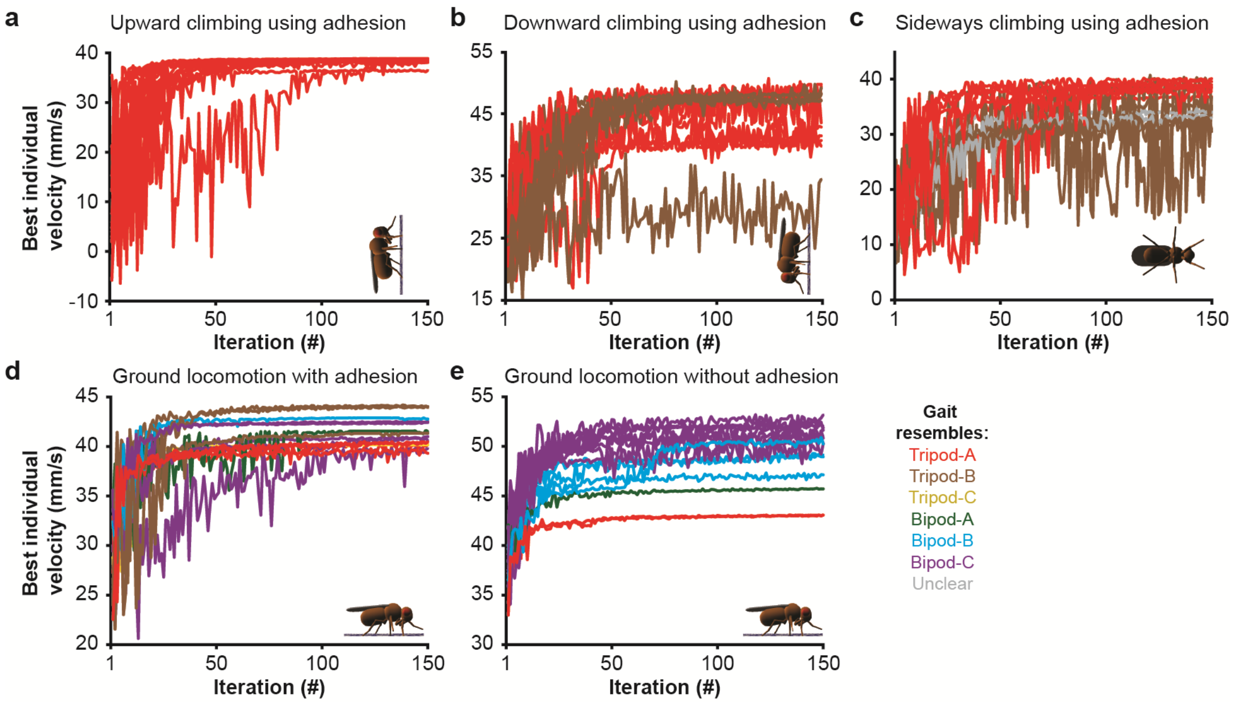

Supplementary Figure 3 | Convergence of fastest forward locomotor velocities during gait optimization. Forward velocities of the fastest individuals for each iteration during gait optimization for forward velocity while (**a**) climbing upward, (**b**) downward, (**c**) or sideways on a vertical surface using leg adhesion, (**d**) walking on the ground with leg adhesion, or (**e**) walking on the ground without leg adhesion. *N* = 15 experiments per condition. Each trace represents a single experiment and is color-coded according to the gait class of the experiment's fastest individual.

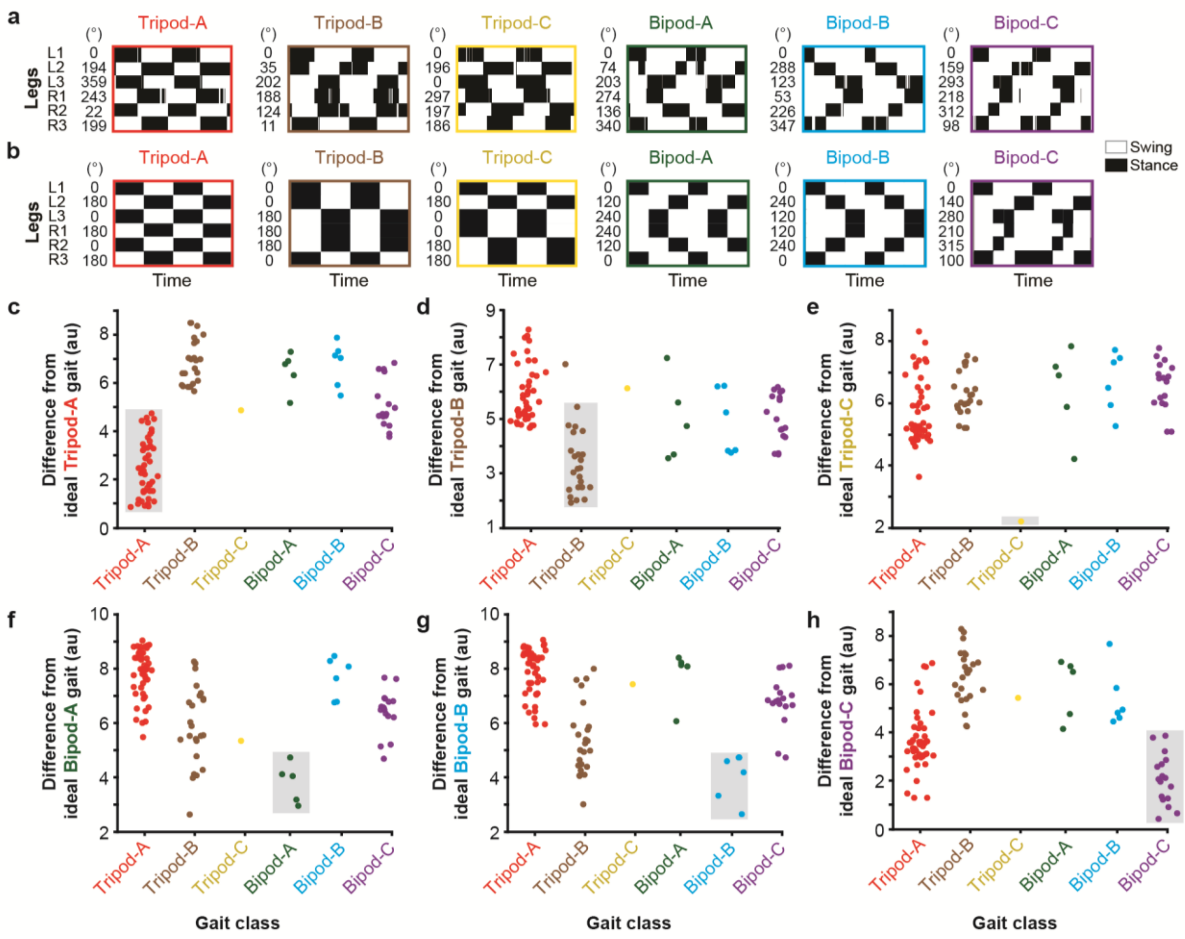

Supplementary Figure 4 | Gait classes and quantitative justification of gait classification. (a) Representative and **(b)** idealized footfall diagrams showing stance (black) and swing (white) phases for each of the six gait classes identified. Two walking cycles are shown for each footfall diagram. The phase of motion for each leg is indicated. **(c-h)** Sum of the difference between leg phases of motion for each optimized gait (sorted by class) versus the idealized **(c)** tripod-A, **(d)** tripod-B, **(e)** tripod-C, **(f)** bipod-A, **(g)** bipod-B, or **(h)** bipod-C gait. Optimized gaits are color-coded by class. Data points are randomly scattered along the x-axis for clarity. Grey boxes highlight optimized gaits within their own, assigned class.

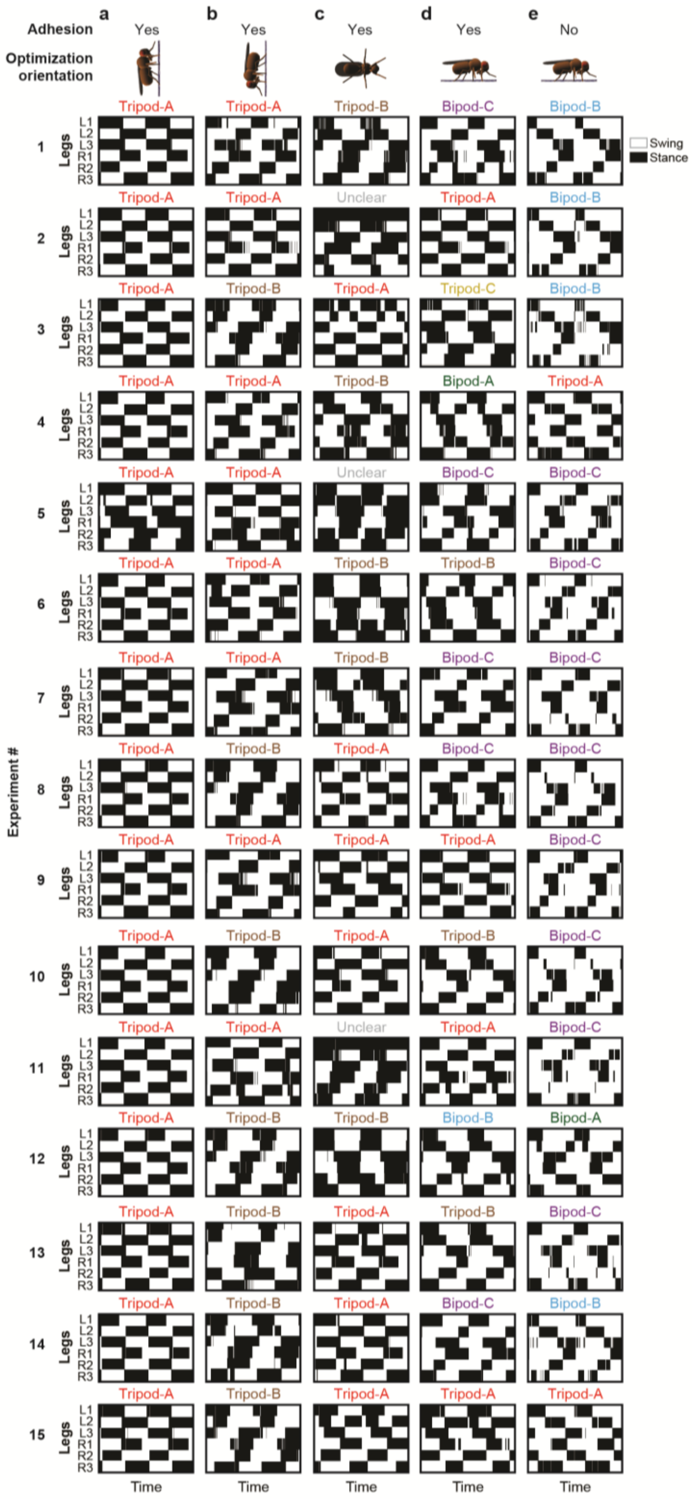

Supplementary Figure 5 | Footfall diagrams for each optimized gait. Footfall diagrams showing stance (black) and swing (white) periods for each experiment. Shown are results for gait optimization of forward velocity while (**a**) climbing upward, (**b**)

climbing downward, (**c**) or climbing sideways on a vertical surface using leg adhesion, (**d**) walking on the ground with leg adhesion, or (**e**) walking on the ground without leg adhesion.

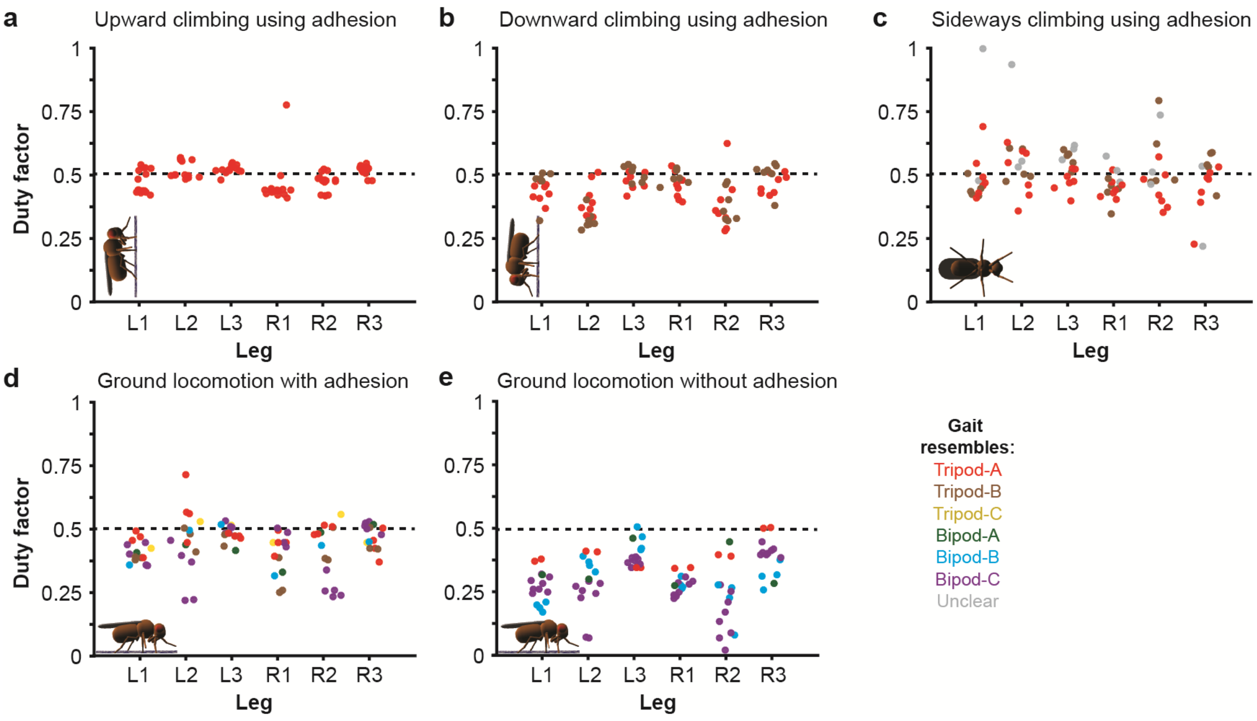

Supplementary Figure 6 | Duty factors for each optimized gait. The duty factor or fraction of time each leg is in contact with the substrate relative to the stride period for all optimized gaits. Shown are duty factors of gaits optimized for (**a**) climbing upward, (**b**) climbing downward, (**c**) or climbing sideways on a vertical surface using leg adhesion, (**d**) walking on the ground with leg adhesion, or (**e**) walking on the ground without leg adhesion. A dashed black line indicates 50% time in contact with the substrate. Optimized gaits are color-coded by class. Data points are randomly scattered along the x-axis for clarity. *N* = 15 for each condition.

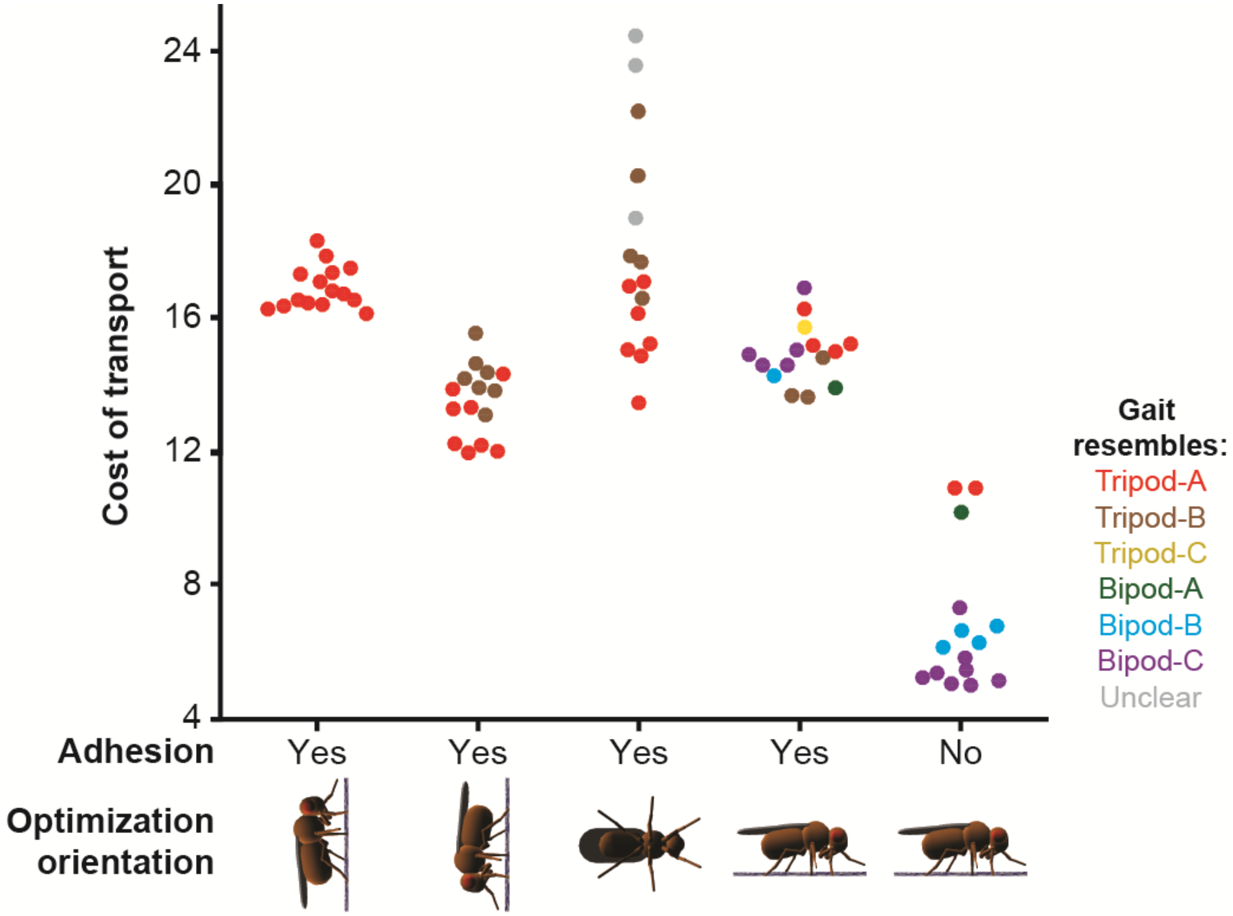

Supplementary Figure 7 | Cost of transport for optimized gaits. The cost of transport (dimensionless) of gaits optimized for forward velocity while climbing upward (left), climbing downward (center-left), or climbing sideways (center) on a vertical surface using leg adhesion, walking on the ground with leg adhesion (center-right), or walking on the ground without leg adhesion (right). Optimized gaits are color-coded by class. Data points are randomly scattered along the x-axis for clarity. *N* = 15 for each condition.

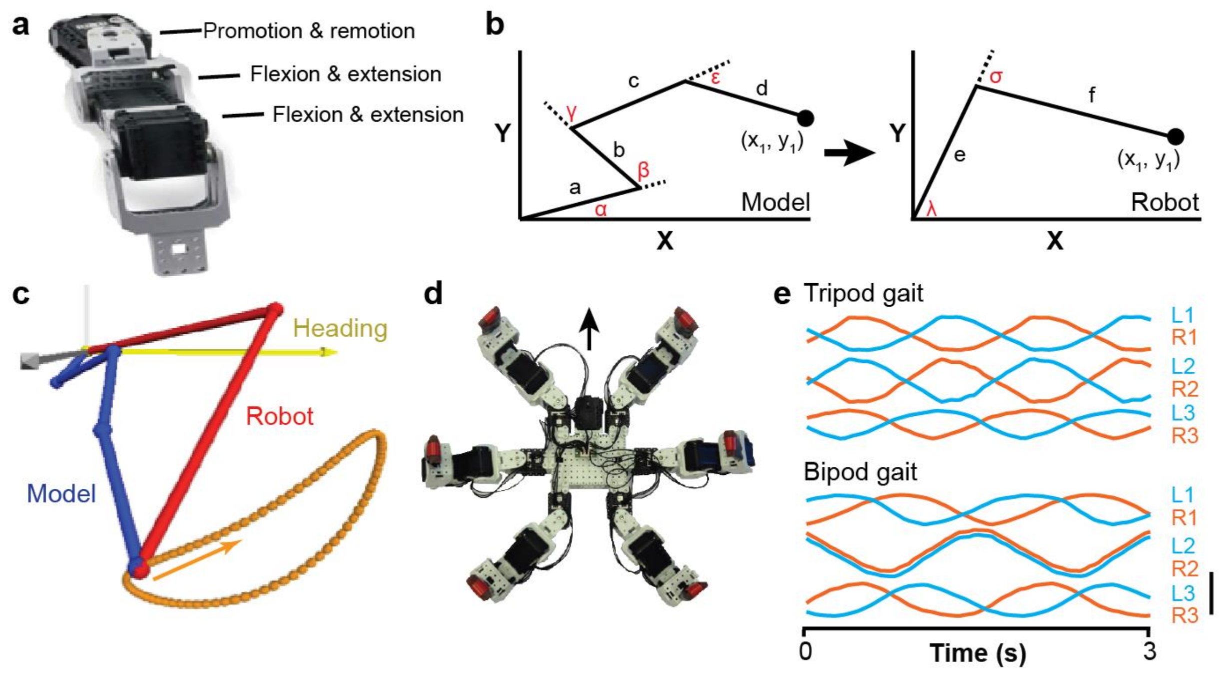

Supplementary Figure 8 | Transferring bipod and tripod gaits to a hexapod robot. **(a)** Image of the robot's leg. Degrees of freedom for each joint are labeled in black text. **(b)** Inverse kinematics approach for mapping the position of the robot's pretarsus (x_1,y_1) to the model's pretarsus despite a reduction from four to two flexion/extension joints. Joint angles are indicated in red. Leg segment lengths are shown in black. **(c)** Visualization of a matched leg trajectory (orange) for the right middle leg pretarsus of the robot (red) and the model (blue). A yellow arrow indicates the direction of heading. **(d)** To track the robot's legs automatically, red tape was affixed to their tips. A black arrow indicates the direction of heading. **(e)** The forward displacement of each of the robot's legs during tripod (top), or bipod-B (bottom) locomotion. Scale bar is 6 cm.

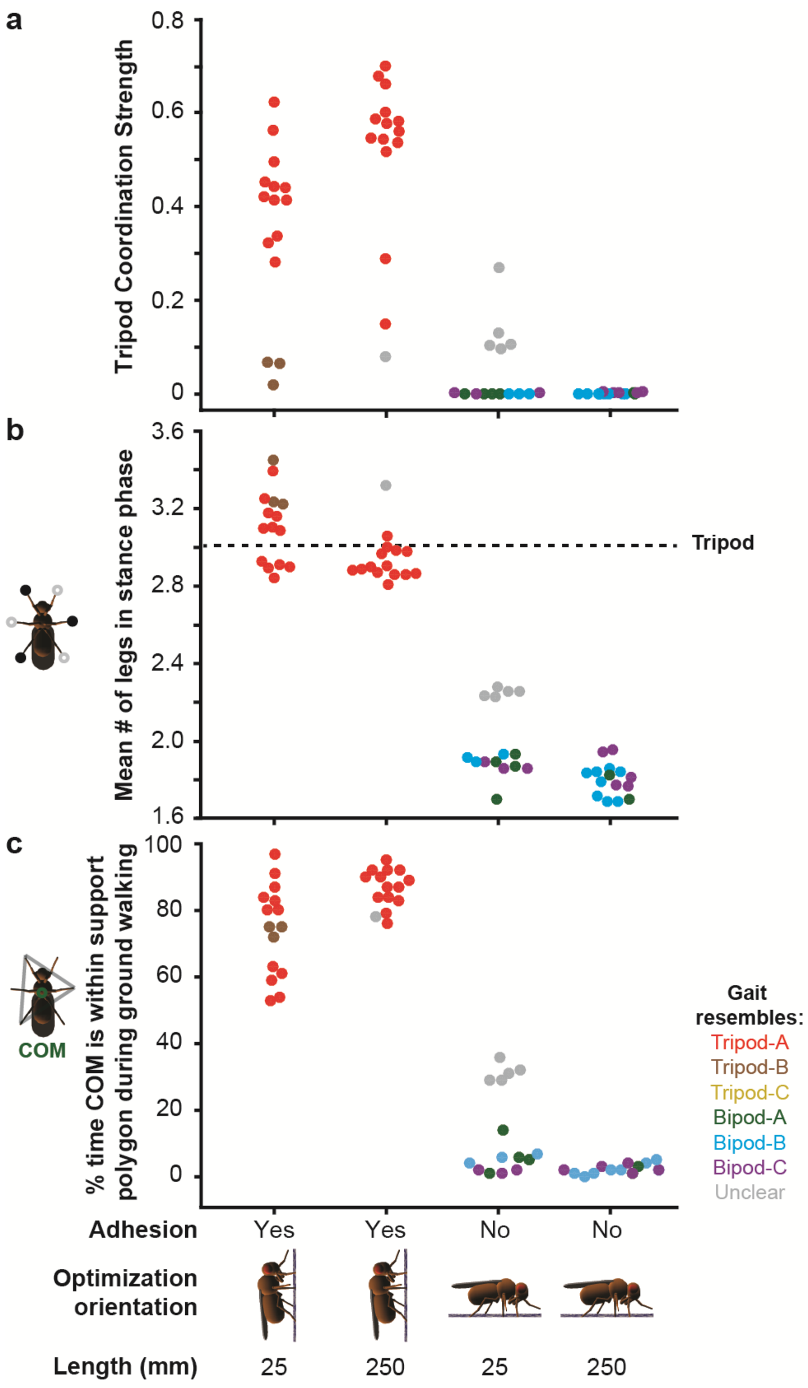

Supplementary Figure 9 | Optimized gaits for models of different sizes. Gaits were optimized for 25 mm, or 250 mm long models for forward velocity while climbing upward (left and middle-left), or walking on the ground without leg adhesion (middle-right and right). **(a)** Tripod Coordination Strength (TCS) values indicating the degree of similarity

to the classic tripod gait footfall diagram (tripod-A). **(b)** The average number of legs in stance phase over five walking cycles. A dashed black line indicates three legs in stance phase as expected for the classic tripod-A gait. **(c)** The percentage of time that the model's center of mass (COM) lies within a polygon of support delineated by each leg in stance phase when the gait is tested during ground walking. Optimized gaits are color-coded by class. Data points are randomly scattered along the x-axis for clarity. N = 15 for each condition.

Body part	Type	Diameter (mm)	Length (mm)	Thickness (mm)	Mass (mg)
Abdomen	Capsule	0.8925	0.595	-	0.0062
Thorax	Sphere	0.952	-	-	0.0124
Head	Capsule	0.595	0.1785	-	0.0124
Wing	Pill-shaped	1.19	1.2495	0.0595	1.236×10^{-5}
Eye	Sphere	0.4165	-	-	(part of head)

Supplementary Table 1 | Geometric dimensions of the model's body. For experiments with larger models (25 mm and 250 mm in length) all dimensions were scaled up while keeping the density of each body part the same.

Body part	Type	Diameter (mm)	Length (mm)	Mass (mg)
Coxa	Capsule	0.1547; 0.1547; 0.1547	0.1547; 0.0952; 0.2737	0.0494
Trochanter/Femur	Capsule	0.1309; 0.1309; 0.1309	0.5653; 0.5177; 0.4879	0.0247
Tibia	Capsule	0.0952; 0.0952; 0.0952	0.5534; 0.4879; 0.4165	0.0247
Tarsus	Capsule	0.0714; 0.0714; 0.0714	0.6069; 0.5415; 0.5355	0.0247
Pretarsus	Sphere	0.119; 0.119; 0.119	-; -; -	0.0124

Supplementary Table 2 | Geometric dimensions of the model's legs (hind/metathoracic; middle/mesothoracic; front/prothoracic). For experiments with larger models (25 mm and 250 mm in length) all dimensions were scaled up while keeping the density of each body part the same.

basicTimeStep	0.2 ms
maxVelocity	100 rad s^{-1}
maxForce (torque for a rotational joint/motor)	2.1 x 10^{-8} Nm
control	50
acceleration	not limited
springConstant	0
dampingConstant	0

Supplementary Table 3 | General and joint parameters values.

Leg	Body-Coxa	Body-Coxa	Body-Coxa	Coxa-Femur	Femur-Tibia	Tibia-Tarsus
Type	promotion/ remotion	abduction/ adduction	rotation	flexion/ extension	flexion/ extension	flexion/ extension
Hind	[-75, -45]	[40, 59]	[-55, -20]	[40, 107.2]	[50, 135]	20.5
Middle	[-25, 25]	25.44	0	[80, 90]	[80, 90]	25.5
Front	[70, 80]	[-40, 10]	[0, 40]	[90, 160]	[55, 125]	21

Supplementary Table 4 | The ranges of motion for each of the model's joints (degrees). Intervals indicate ranges, single values indicate constant position without oscillation.

Leg	Body-Coxa	Body-Coxa	Body-Coxa	Coxa-Femur	Femur-Tibia	Tibia-Tarsus
Type	promotion/remotion	abduction/adduction	rotation	flexion/extension	flexion/extension	flexion/extension
Hind	180	0	180	200	180	0
Middle	180	0	0	270	90	0
Front	0	210	0	0	20	0

Supplementary Table 5 | The relative phase of oscillation for each of the model's joints (degrees).
